# Bis(*O*-ethyl dithio­carbonato-κ^2^ *S*,*S*′)bis­(pyridine-3-carbonitrile-κ*N* ^1^)nickel(II)

**DOI:** 10.1107/S1600536811053475

**Published:** 2011-12-17

**Authors:** Sanjay Kapoor, Ramandeep Kour, Renu Sachar, Rajni Kant, Vivek K. Gupta, Kamini Kapoor

**Affiliations:** a Department of Chemistry, University of Jammu, Jammu Tawi 180 006, India; b X-ray Crystallography Laboratory, Post-Graduate Department of Physics & Electronics, University of Jammu, Jammu Tawi 180 006, India

## Abstract

The Ni^2+^ ion in the title complex, [Ni(C_3_H_5_OS_2_)_2_(C_6_H_4_N_2_)_2_], is in a strongly distorted octa­hedral coordination environment formed by an N_2_S_4_ donor set, with the Ni^2+^ ion located on a centre of inversion. In the crystal, weak C—H⋯S and C—H⋯N inter­actions are observed.

## Related literature

For related structures, see: Tiekink & Haiduc (2005 ▶); Dakternieks *et al.* (2006 ▶); Hill & Tiekink (2007 ▶); Hogarth *et al.* (2009 ▶)
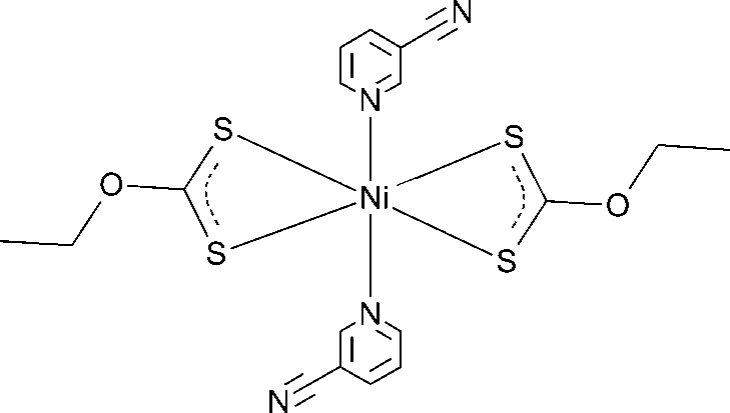


## Experimental

### 

#### Crystal data

[Ni(C_3_H_5_OS_2_)_2_(C_6_H_4_N_2_)_2_]
*M* *_r_* = 509.31
Monoclinic, 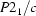

*a* = 6.7302 (2) Å
*b* = 18.8959 (5) Å
*c* = 8.7242 (2) Å
β = 95.916 (2)°
*V* = 1103.58 (5) Å^3^
*Z* = 2
Mo *K*α radiation
μ = 1.28 mm^−1^
*T* = 293 K
0.3 × 0.3 × 0.1 mm

#### Data collection

Oxford Diffraction Xcalibur Sapphire3 diffractometer
Absorption correction: multi-scan (*CrysAlis PRO*; Oxford Diffraction, 2007 ▶) *T*_min_ = 0.728, *T*_max_ = 1.000
18847 measured reflections
1930 independent reflections
1723 reflections with *I* > 2σ(*I*)
*R*_int_ = 0.036

#### Refinement

*R*[*F* ^2^ > 2σ(*F* ^2^)] = 0.025
*wR*(*F* ^2^) = 0.060
*S* = 1.07
1930 reflections
134 parameters
H-atom parameters constrained
Δρ_max_ = 0.23 e Å^−3^
Δρ_min_ = −0.22 e Å^−3^

### 

Data collection: *CrysAlis PRO* (Oxford Diffraction, 2007 ▶); cell refinement: *CrysAlis PRO*; data reduction: *CrysAlis PRO*; program(s) used to solve structure: *SHELXS97* (Sheldrick, 2008 ▶); program(s) used to refine structure: *SHELXL97* (Sheldrick, 2008 ▶); molecular graphics: *ORTEP-3* (Farrugia, 1997 ▶); software used to prepare material for publication: *PLATON* (Spek, 2009 ▶) and *PARST* (Nardelli, 1995 ▶).

## Supplementary Material

Crystal structure: contains datablock(s) I, global. DOI: 10.1107/S1600536811053475/gk2438sup1.cif

Structure factors: contains datablock(s) I. DOI: 10.1107/S1600536811053475/gk2438Isup2.hkl

Additional supplementary materials:  crystallographic information; 3D view; checkCIF report

## Figures and Tables

**Table 1:** Selected bond lengths (Å)

Ni1—N1	2.1273 (17)
Ni1—S1	2.4335 (5)
Ni1—S2	2.4450 (6)

**Table 2:** Hydrogen-bond geometry (Å, °)

*D*—H⋯*A*	*D*—H	H⋯*A*	*D*⋯*A*	*D*—H⋯*A*
C9—H9*A*⋯S2^i^	0.97	2.85	3.455 (2)	121
C10—H10*C*⋯N2^ii^	0.96	2.65	3.595 (4)	169

Symmetry codes: (i) 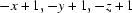; (ii) 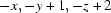.

**Fig. 1.**
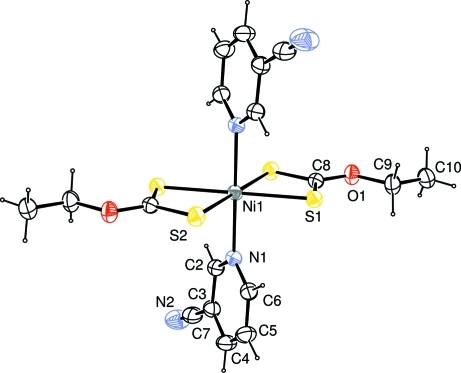
ORTEP view of the molecule with displacement ellipsoids drawn at the 30% probability level. Unlabeled atoms are generated by the symmetry operation -x, 1-y, 1-z.

**Fig. 2.**
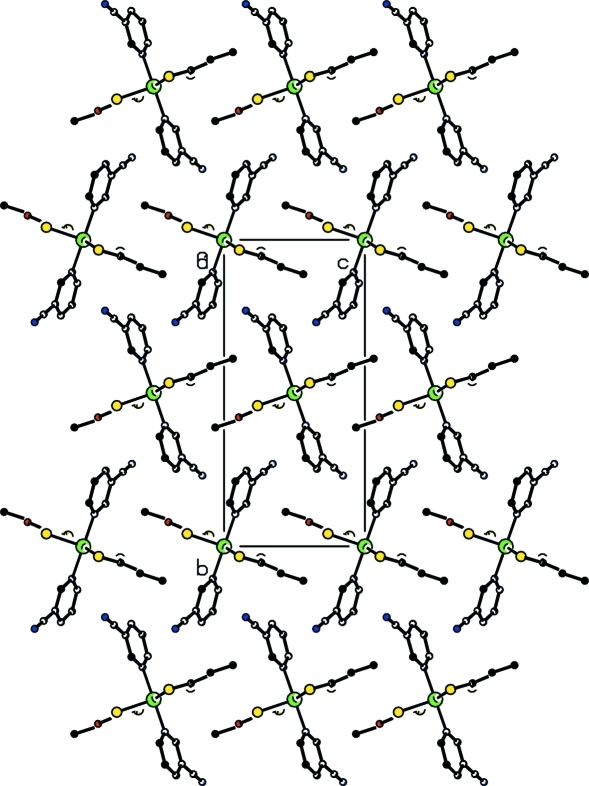
The packing arrangement of molecules viewed down the a axis.

## supplementary crystallographic information

### Comment

Xanthates (*O*-alkyl/aryl dithiocarbonates) have been known for a long time and many adducts of metal xanthates with different ligands have been prepared and studied in the last several decades. Adducts of transition metal xanthates with N-donor ligands are well represented in the literature, the most extensively studied being those of nickel(II). Nitrogen containing adducts of nickel(II) xanthates are known to adopt a variety of supramolecular assemblies (Tiekink & Haiduc, 2005). The Ni atom in (I) is located on a center of inversion and exists within a *trans*-N2S4 donor set that defines an approximately octahedral coordination geometry. The chelating xanthate ligand forms essentially equivalent Ni—S bond distances; this equivalence is reflected in the parameters defining the xanthate ligand. The bond angles around the Ni atom are in the range of 73.83 (2) to 180.00 (3)°. The Ni—S bond lengths, Ni1—S1 = 2.4335 (5); Ni1—S2 = 2.4450 (6) Å, are in good agreement with those reported for other Ni-dithiocarbonato complexes (Tiekink & Haiduc, 2005; Dakternieks *et al.*, 2006; Hill & Tiekink, 2007; Hogarth *et al.*, 2009). Molecules in the unit cell are packed together to form well defined layers. While no classical hydrogen bonds are present, the C—H···S and C—H···N hydrogen bonds (Table 1) play important role in stabilizing the crystal structure.

### Experimental

The title complex was prepared by stirring the parent nickel(II) ethylxanthate (0.781g,0.0026 mol.) with 3-cyanopyridine(0.541g, 0.0052 mol.) in acetone(60 ml) for one hour. Green crystals of (I) were isolated by the slow evaporation of the resulting solution of the complex.

### Refinement

All H atoms were positioned geometrically and were treated as riding on their parent C atoms, with C—H distances of 0.93–0.97 Å and with *U*_iso_(H) = 1.2Ueq(C) or 1.5Ueq(methylC).

### Crystal data

**Table:**

[Ni(C_3_H_5_OS_2_)_2_(C_6_H_4_N_2_)_2_]	*F*(000) = 524
*M**_r_* = 509.31	*D*_x_ = 1.533 Mg m^−^^3^
Monoclinic, *P*2_1_/*c*	Mo *K*α radiation, λ = 0.71073 Å
Hall symbol: -P 2ybc	Cell parameters from 10361 reflections
*a* = 6.7302 (2) Å	θ = 3.6–29.0°
*b* = 18.8959 (5) Å	µ = 1.28 mm^−^^1^
*c* = 8.7242 (2) Å	*T* = 293 K
β = 95.916 (2)°	Hexagonal plate, green
*V* = 1103.58 (5) Å^3^	0.3 × 0.3 × 0.1 mm
*Z* = 2	

### Data collection

**Table:**

Oxford Diffraction Xcalibur Sapphire3 diffractometer	1930 independent reflections
Radiation source: fine-focus sealed tube	1723 reflections with *I* > 2σ(*I*)
graphite	*R*_int_ = 0.036
Detector resolution: 16.1049 pixels mm^-1^	θ_max_ = 25.0°, θ_min_ = 3.7°
ω scans	*h* = −8→8
Absorption correction: multi-scan (*CrysAlis PRO*; Oxford Diffraction, 2007)	*k* = −22→22
*T*_min_ = 0.728, *T*_max_ = 1.000	*l* = −10→10
18847 measured reflections	

### Refinement

**Table:**

Refinement on *F*^2^	Primary atom site location: structure-invariant direct methods
Least-squares matrix: full	Secondary atom site location: difference Fourier map
*R*[*F*^2^ > 2σ(*F*^2^)] = 0.025	Hydrogen site location: inferred from neighbouring sites
*wR*(*F*^2^) = 0.060	H-atom parameters constrained
*S* = 1.07	*w* = 1/[σ^2^(*F*_o_^2^) + (0.0208*P*)^2^ + 0.7659*P*] where *P* = (*F*_o_^2^ + 2*F*_c_^2^)/3
1930 reflections	(Δ/σ)_max_ = 0.001
134 parameters	Δρ_max_ = 0.23 e Å^−^^3^
0 restraints	Δρ_min_ = −0.22 e Å^−^^3^

### Special details

**Table:**

Geometry. All e.s.d.'s (except the e.s.d. in the dihedral angle between two l.s. planes) are estimated using the full covariance matrix. The cell e.s.d.'s are taken into account individually in the estimation of e.s.d.'s in distances, angles and torsion angles; correlations between e.s.d.'s in cell parameters are only used when they are defined by crystal symmetry. An approximate (isotropic) treatment of cell e.s.d.'s is used for estimating e.s.d.'s involving l.s. planes.
Refinement. Refinement of *F*^2^ against ALL reflections. The weighted *R*-factor *wR* and goodness of fit *S* are based on *F*^2^, conventional *R*-factors *R* are based on *F*, with *F* set to zero for negative *F*^2^. The threshold expression of *F*^2^ > σ(*F*^2^) is used only for calculating *R*-factors(gt) *etc*. and is not relevant to the choice of reflections for refinement. *R*-factors based on *F*^2^ are statistically about twice as large as those based on *F*, and *R*- factors based on ALL data will be even larger.

### Fractional atomic coordinates and isotropic or equivalent isotropic displacement parameters (Å^2^)

**Table:**

	*x*	*y*	*z*	*U*_iso_*/*U*_eq_	
Ni1	0.0000	0.5000	0.5000	0.02943 (12)	
S1	0.33453 (8)	0.46692 (3)	0.60894 (6)	0.03356 (14)	
S2	0.03241 (8)	0.54100 (3)	0.23794 (7)	0.03737 (15)	
O1	0.3099 (2)	0.42097 (8)	0.89474 (17)	0.0404 (4)	
N1	0.0499 (3)	0.60555 (9)	0.5796 (2)	0.0340 (4)	
N2	−0.3300 (4)	0.76898 (12)	0.8455 (3)	0.0691 (7)	
C2	−0.0808 (3)	0.63914 (11)	0.6579 (3)	0.0368 (5)	
H2	−0.1910	0.6143	0.6856	0.044*	
C3	−0.0591 (3)	0.71000 (12)	0.6999 (3)	0.0401 (5)	
C4	0.1050 (4)	0.74716 (13)	0.6592 (3)	0.0484 (6)	
H4	0.1222	0.7947	0.6846	0.058*	
C5	0.2413 (4)	0.71212 (13)	0.5805 (3)	0.0469 (6)	
H5	0.3542	0.7354	0.5532	0.056*	
C6	0.2086 (3)	0.64182 (12)	0.5425 (3)	0.0383 (5)	
H6	0.3014	0.6186	0.4885	0.046*	
C7	−0.2097 (4)	0.74323 (13)	0.7818 (3)	0.0497 (6)	
C8	0.2162 (3)	0.44638 (11)	0.7649 (2)	0.0319 (5)	
C9	0.5252 (3)	0.41173 (13)	0.9032 (3)	0.0446 (6)	
H9A	0.5889	0.4561	0.8807	0.054*	
H9B	0.5583	0.3767	0.8285	0.054*	
C10	0.5962 (4)	0.38776 (14)	1.0625 (3)	0.0520 (6)	
H10A	0.5554	0.4213	1.1357	0.078*	
H10B	0.7392	0.3842	1.0732	0.078*	
H10C	0.5395	0.3423	1.0810	0.078*	

### Atomic displacement parameters (Å^2^)

**Table:**

	*U*^11^	*U*^22^	*U*^33^	*U*^12^	*U*^13^	*U*^23^
Ni1	0.0264 (2)	0.0265 (2)	0.0358 (2)	0.00092 (15)	0.00515 (15)	0.00119 (16)
S1	0.0274 (3)	0.0369 (3)	0.0373 (3)	0.0017 (2)	0.0078 (2)	0.0051 (2)
S2	0.0275 (3)	0.0441 (3)	0.0418 (3)	0.0007 (2)	0.0097 (2)	0.0045 (2)
O1	0.0312 (8)	0.0509 (10)	0.0392 (9)	0.0036 (7)	0.0040 (7)	0.0093 (7)
N1	0.0334 (10)	0.0296 (9)	0.0387 (10)	0.0001 (8)	0.0028 (8)	0.0021 (8)
N2	0.0700 (17)	0.0489 (14)	0.0917 (19)	0.0032 (12)	0.0249 (14)	−0.0198 (13)
C2	0.0360 (12)	0.0332 (12)	0.0412 (13)	−0.0003 (9)	0.0035 (10)	−0.0004 (10)
C3	0.0443 (13)	0.0338 (12)	0.0412 (13)	0.0033 (10)	0.0001 (10)	−0.0040 (10)
C4	0.0590 (16)	0.0314 (12)	0.0535 (15)	−0.0084 (11)	−0.0004 (12)	−0.0019 (11)
C5	0.0466 (14)	0.0411 (13)	0.0531 (15)	−0.0124 (11)	0.0061 (11)	0.0036 (12)
C6	0.0351 (12)	0.0371 (12)	0.0424 (13)	−0.0012 (9)	0.0033 (10)	0.0038 (10)
C7	0.0561 (16)	0.0361 (13)	0.0565 (16)	−0.0024 (12)	0.0042 (13)	−0.0096 (12)
C8	0.0306 (11)	0.0294 (11)	0.0357 (11)	−0.0003 (9)	0.0028 (9)	0.0021 (9)
C9	0.0341 (12)	0.0503 (14)	0.0488 (14)	0.0072 (10)	0.0013 (10)	0.0061 (11)
C10	0.0525 (15)	0.0469 (15)	0.0536 (15)	0.0075 (12)	−0.0082 (12)	0.0037 (12)

### Geometric parameters (Å, °)

**Table:**

Ni1—N1	2.1273 (17)	C3—C7	1.442 (3)
Ni1—S1	2.4335 (5)	C4—C5	1.372 (3)
Ni1—S2	2.4450 (6)	C4—H4	0.9300
S1—C8	1.691 (2)	C5—C6	1.381 (3)
S2—C8^i^	1.688 (2)	C5—H5	0.9300
O1—C8	1.328 (2)	C6—H6	0.9300
O1—C9	1.454 (3)	C9—C10	1.493 (3)
N1—C2	1.329 (3)	C9—H9A	0.9700
N1—C6	1.336 (3)	C9—H9B	0.9700
N2—C7	1.138 (3)	C10—H10A	0.9600
C2—C3	1.392 (3)	C10—H10B	0.9600
C2—H2	0.9300	C10—H10C	0.9600
C3—C4	1.385 (3)		
			
N1^i^—Ni1—N1	180.00 (3)	C2—C3—C7	119.3 (2)
N1^i^—Ni1—S1	89.73 (5)	C5—C4—C3	118.4 (2)
N1—Ni1—S1	90.27 (5)	C5—C4—H4	120.8
N1^i^—Ni1—S1^i^	90.27 (5)	C3—C4—H4	120.8
N1—Ni1—S1^i^	89.73 (5)	C4—C5—C6	119.1 (2)
S1—Ni1—S1^i^	180.0	C4—C5—H5	120.5
N1^i^—Ni1—S2^i^	88.96 (5)	C6—C5—H5	120.5
N1—Ni1—S2^i^	91.04 (5)	N1—C6—C5	123.1 (2)
S1—Ni1—S2^i^	73.831 (18)	N1—C6—H6	118.4
S1^i^—Ni1—S2^i^	106.169 (18)	C5—C6—H6	118.4
N1^i^—Ni1—S2	91.04 (5)	N2—C7—C3	179.3 (3)
N1—Ni1—S2	88.96 (5)	O1—C8—S2^i^	116.53 (15)
S1—Ni1—S2	106.169 (18)	O1—C8—S1	123.21 (15)
S1^i^—Ni1—S2	73.831 (18)	S2^i^—C8—S1	120.26 (12)
S2^i^—Ni1—S2	180.0	O1—C9—C10	107.76 (19)
C8—S1—Ni1	83.10 (7)	O1—C9—H9A	110.2
C8^i^—S2—Ni1	82.80 (7)	C10—C9—H9A	110.2
C8—O1—C9	118.02 (17)	O1—C9—H9B	110.2
C2—N1—C6	117.86 (19)	C10—C9—H9B	110.2
C2—N1—Ni1	121.65 (14)	H9A—C9—H9B	108.5
C6—N1—Ni1	120.33 (15)	C9—C10—H10A	109.5
N1—C2—C3	122.5 (2)	C9—C10—H10B	109.5
N1—C2—H2	118.7	H10A—C10—H10B	109.5
C3—C2—H2	118.7	C9—C10—H10C	109.5
C4—C3—C2	119.0 (2)	H10A—C10—H10C	109.5
C4—C3—C7	121.7 (2)	H10B—C10—H10C	109.5
			
N1^i^—Ni1—S1—C8	−88.58 (9)	C6—N1—C2—C3	0.8 (3)
N1—Ni1—S1—C8	91.42 (9)	Ni1—N1—C2—C3	−174.57 (16)
S2^i^—Ni1—S1—C8	0.42 (7)	N1—C2—C3—C4	−0.1 (3)
S2—Ni1—S1—C8	−179.58 (7)	N1—C2—C3—C7	178.6 (2)
N1^i^—Ni1—S2—C8^i^	90.39 (9)	C2—C3—C4—C5	−0.9 (4)
N1—Ni1—S2—C8^i^	−89.61 (9)	C7—C3—C4—C5	−179.6 (2)
S1—Ni1—S2—C8^i^	−179.58 (7)	C3—C4—C5—C6	1.2 (4)
S1^i^—Ni1—S2—C8^i^	0.42 (7)	C2—N1—C6—C5	−0.5 (3)
S1—Ni1—N1—C2	−130.63 (16)	Ni1—N1—C6—C5	174.93 (18)
S1^i^—Ni1—N1—C2	49.37 (16)	C4—C5—C6—N1	−0.5 (4)
S2^i^—Ni1—N1—C2	−56.80 (16)	C9—O1—C8—S2^i^	−177.93 (16)
S2—Ni1—N1—C2	123.20 (16)	C9—O1—C8—S1	1.2 (3)
S1—Ni1—N1—C6	54.10 (16)	Ni1—S1—C8—O1	−179.72 (18)
S1^i^—Ni1—N1—C6	−125.90 (16)	Ni1—S1—C8—S2^i^	−0.67 (12)
S2^i^—Ni1—N1—C6	127.93 (16)	C8—O1—C9—C10	176.01 (19)
S2—Ni1—N1—C6	−52.07 (16)		

Symmetry codes: (i) −*x*, −*y*+1, −*z*+1.

### Hydrogen-bond geometry (Å, °)

**Table:**

*D*—H···*A*	*D*—H	H···*A*	*D*···*A*	*D*—H···*A*
C9—H9A···S2^ii^	0.97	2.85	3.455 (2)	121
C10—H10C···N2^iii^	0.96	2.65	3.595 (4)	169

Symmetry codes: (ii) −*x*+1, −*y*+1, −*z*+1; (iii) −*x*, −*y*+1, −*z*+2.
